# Peer effects on control-averse behavior

**DOI:** 10.1038/s41598-019-39600-9

**Published:** 2019-02-28

**Authors:** Sarah Rudorf, Thomas Baumgartner, Daria Knoch

**Affiliations:** Department of Social Psychology and Social Neuroscience, Institute of Psychology, University of Bern, 3012 Bern, Switzerland

## Abstract

The urge to rebel against external control affects social interactions in many domains of our society with potentially far-reaching consequences. Nevertheless, it has remained unclear to what degree this control-averse behavior might be influenced by the people in our surroundings, our peers. In an experimental paradigm with real restrictions of the subjects’ freedom of choice and no systematic incentives to follow the peer, we are able to demonstrate both negative and positive peer effects on control-averse behavior. First, we find that information about a peer’s strongly control-averse behavior, although irrelevant for the subjects’ outcome, increases the subjects’ individual control-averse behavior. Second, we find that information about a peer’s more generous and only weakly control-averse behavior increases subjects’ generous behavior, even though it is associated with greater costs for the subjects. Critically, each subject’s behavior determined the monetary payoff of both the subject and a third person, thereby constituting a social behavior with actual consequences. Interestingly, these peer effects are not moderated by self-assessments of the general resistance to peer influence or the general tendency to rebel against restrictions of one’s freedom of choice. Contributing new insights into a complex and highly relevant social phenomenon, our results indicate that information about a single peer’s behavior can influence individual control-averse behavior.

## Introduction

Control-averse behavior, that is the negative response to exogenous control over one’s decisions, is a prevalent phenomenon in our society. It can involve disobedience of employees^1^, noncompliance of patients in psychotherapy^2^ or teenage rebellion against disciplinary measures^3^, and therefore places a burden on economic, health and social systems.

Recent work has improved our understanding of control-averse behavior by providing first insights into the underlying neural processes and the functional brain organization^4,5^. Whereas these studies have illuminated the neurobiological basis of individual differences in control-averse behavior, the current study goes one step further and asks whether individual control-averse behavior can be influenced by external factors. Understanding what influences and shapes control-averse behavior could help prevent negative consequences and is thus an important research goal. In particular, it remains to be investigated whether control-averse behavior might be influenced by the people around us, our peers.

Peer influence is a strong determinant of social behavior. This has been shown in studies using hypothetical scenarios or questionnaires^6–8^ as well as field experiments^9,10^. Controlled experiments that measure actual social behavior with real consequences, however, remain rare^11–15^. Of the few studies assessing actual (not hypothetical) social behavior, some have investigated the effects of peer feedback on prosocial behavior^11,16^, whereas others have focused on the effects of information about peer behavior. For example, Nook *et al*.^12^ found that information about monetary donations made by peers led participants to adjust their donations to match those of the peers. Moreover, Park and Shin^14^ recently showed that the observation of prosocial peer behavior increases charitable donations, whereas Thöni and Gächter^13^ showed that information about uncooperative peer behavior can diminish participants’ cooperative behavior. Together, these studies demonstrate that peer influence can have strong effects on social behavior. To date, however, no study has investigated the effects of peer influence on control-averse behavior.

The current study aims to close this gap and test whether information about a peer’s control-averse behavior can modulate individual control-averse behavior. To this end, we implemented a novel experimental paradigm in which peer influence was manipulated between subjects, and control-averse behavior was measured within subjects. Concretely, control-averse behavior was assessed as the subjects’ behavioral responses to real restrictions of their freedom of choice by other participants. Peer influence, on the other hand, was implemented as the repeated information about a peer’s behavioral responses in the same situations. Each subject observed either a strongly control-averse peer or a weakly control-averse peer. The peer’s behavior was modeled after participants with similar demographics who had participated in a previous study. For comparison, we added a separate experimental group without peer influence. We predicted that, in comparison to the group without peer influence, observing a strongly control-averse peer would increase the subjects’ control-averse behavior, whereas observing a weakly control-averse peer would decrease the subjects’ control-averse behavior. Previous work has suggested that peer effects might be moderated by a general resistance to peer influence^17^ or the general motivation to rebel against control^18^. To account for these individual characteristics, we assessed them using standardized questionnaires^17,19,20^. The results provide novel insights into peer effects on social behavior and bear implications for neuroscientific investigations as well as clinical work.

## Results

### Control-averse behavior without peer influence

**Figure 1:**
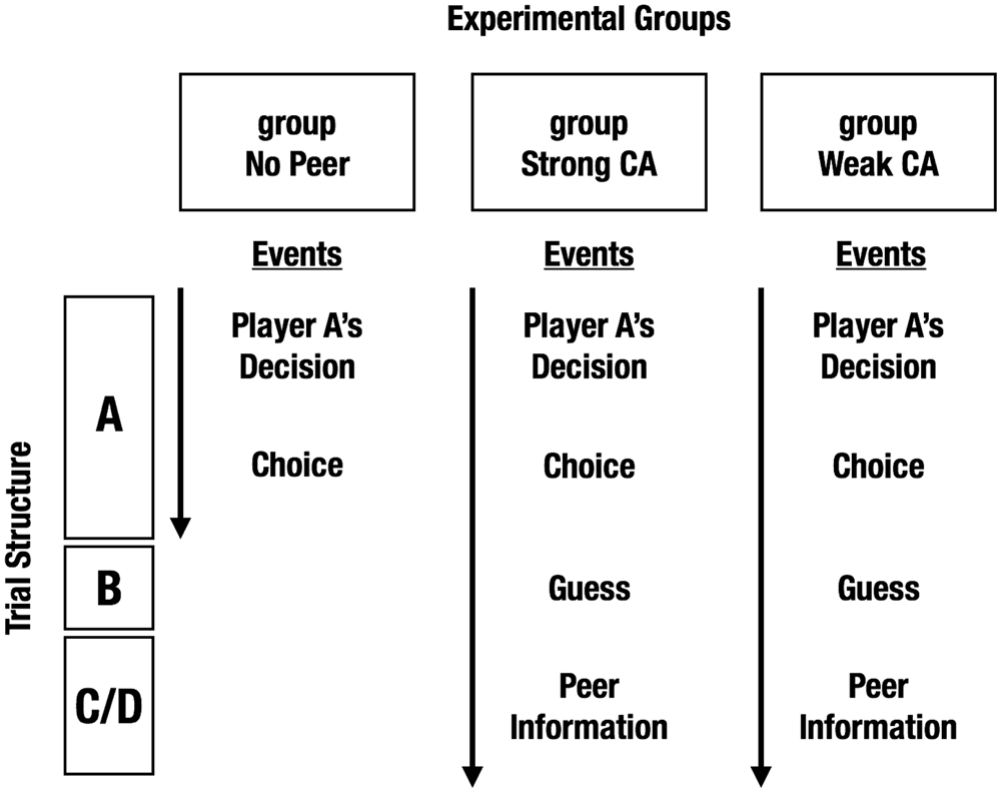
Experimental groups and respective trial structures of the Control aversion task. Each of three experimental groups completed a different version of the Control aversion task. The letters (**A**–**D**) refer to panels in Fig. 2. Subjects in the group No Peer first see whether player A lets them choose freely (Free condition) or controls their choice options (Controlled condition). Then they make a choice, and the next trial begins. Subjects in the groups Strong CA and Weak CA first see whether player A lets them choose freely or controls their choice options. Then they make a choice. In one third of all trials, the next trial begins; in two thirds of all trials, the subjects then guess what the peer (player B*) has chosen in the same situation. Then they see what the peer has chosen: subjects in the group Strong CA receive information about a strongly control-averse peer, whereas subjects in the group Weak CA receive information about a weakly control-averse peer. Then the next trial begins. CA, control aversion.

**Figure 2:**
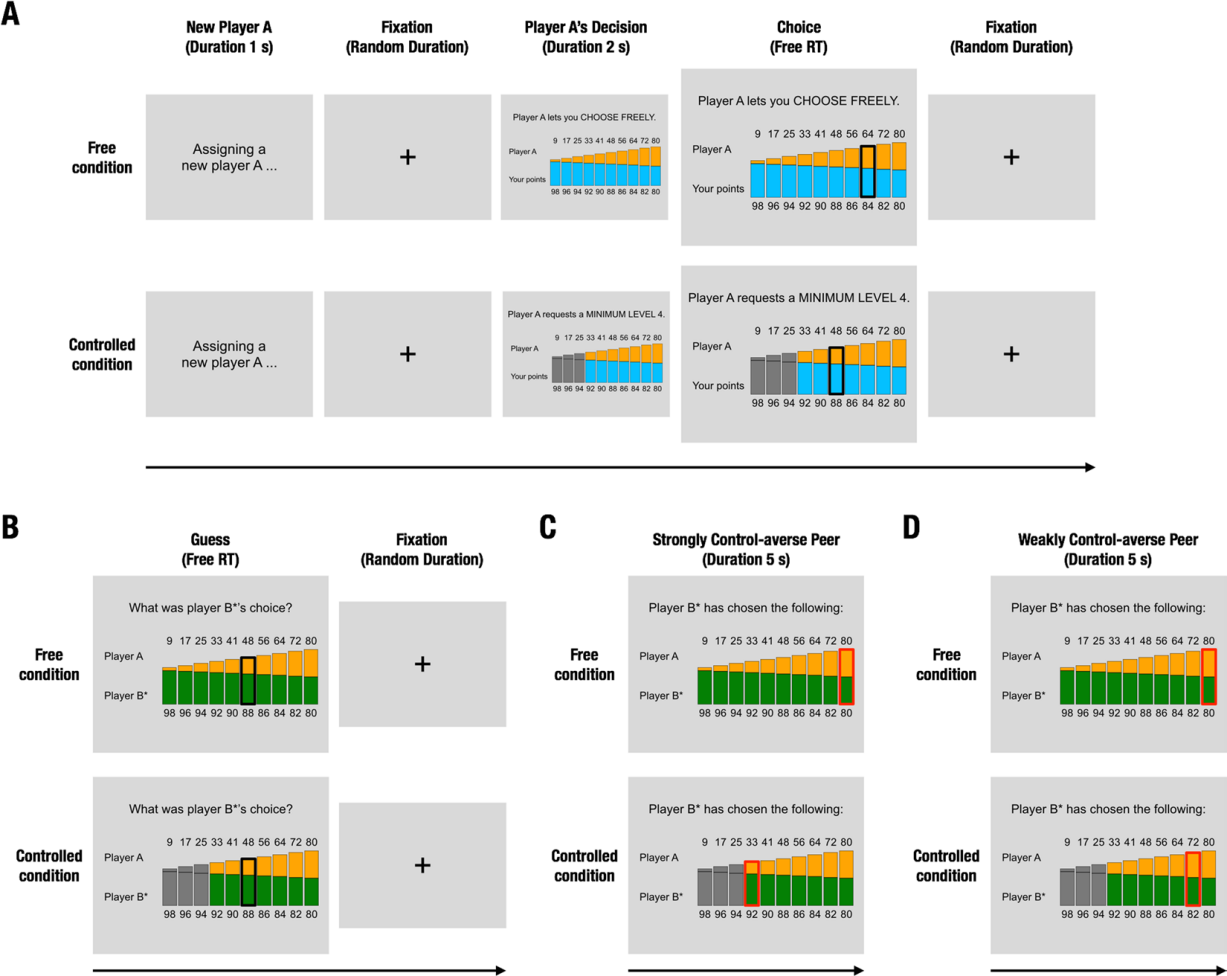
Control aversion task with peer influence. (**A**), implementation of the choice restriction. In every trial, a new player A either lets the subject choose freely (Free condition) or requests a minimum of level four (Controlled condition). After a delay of 2 seconds, the subject chooses a level by moving a black frame. For trials without peer information and subjects in the group No Peer, the trial ends here. (**B**–**D**), implementation of the peer influence. In two thirds of the trials, subjects in the groups with peer information then guess what a peer (player B*) chose in the same situation by moving a black frame (**B**). Then subjects see the peer’s actual choice: Subjects in the group Strong CA observe the choice by a strongly control-averse peer (**C**), whereas subjects in the group Weak CA observe the choice by a weakly control-averse peer (**D**), which is indicated by a red frame. For each group, the peer remains identical throughout the experiment. CA, control aversion; RT, Reaction Time.

Data from 82 subjects were analyzed in this study (42 women, 40 men, *M*_*age*_ = 22 ± 3 *SD* years). Twenty-three of those subjects were assigned to an experimental group without peer influence (group No Peer, Figs 1 and 2). To assess these subjects’ levels of control-averse behavior, we implemented a Control aversion task, in which subjects made choices under two conditions (Fig. 2A): In the Free condition, subjects could choose freely among ten allocation options, called generosity levels, ranging from selfish to more generous monetary allocations between themselves and another person. In the Controlled condition, the other person requested a minimum of generosity and thereby eliminated the three most selfish options. Importantly, one allocation choice per subject was randomly selected at the end of the experiment and paid out to the subject as well as the respective other person. This was done to motivate subjects to behave according to their true preferences in all trials.

**Figure 3:**
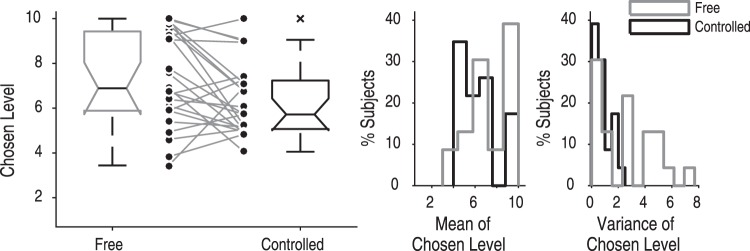
Control-averse behavior in the group without peer information (group No Peer, *n* = 23). On average, subjects chose lower levels in the Controlled condition than in the Free condition, thereby displaying control-averse behavior. Left, boxplots of the chosen levels in the Free and the Controlled condition. The central mark of each box shows the median, the box edges show the 25th and 75th percentiles, the notches of each box depict the 95% confidence intervals of the median, and the whiskers represent the limit beyond which a data point is considered an outlier (displayed as a cross). The connected data points in the center show individual subjects’ means. Right, histograms showing the distributions of subjects’ means and variances of chosen levels in the Free and the Controlled condition.

We first analyzed the choice behavior of the group No Peer, who completed the Control aversion task without peer influence. In line with previous work^4^, subjects in the No Peer group chose, on average, lower levels in the Controlled condition (*M* = 6.38 ± 1.63 *SD*, *Mdn* = 5.72) than in the Free condition (*M* = 7.52 ± 1.93 *SD*, *Mdn* = 7.22; Wilcoxon signed rank test, one-tailed, *z* = −2.59, *p* = 0.005, effect size *r* = −0.38; Hodges-Lehmann median of differences = 1.07, 95% CI [0.28, 1.92]; Fig. 3). Note, that the statistical test was corrected for a bottom effect, following the procedure by Falk and Kosfeld^1^ (see Methods for details).

**Figure 4:**
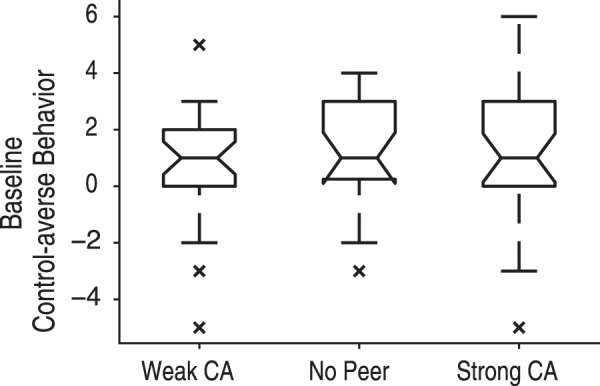
Control-averse behavior at baseline. At baseline (i.e. in the first two trials prior to any peer information), subjects across all groups displayed a similar level of control-averse behavior as measured by the chosen level in the Free trial minus the chosen level in the Controlled trial. The central mark of each box shows the median, the box edges show the 25th and 75th percentiles, and the notches of each box depict the 95% confidence intervals of the median. The whiskers represent the limit beyond which data points are considered outliers, which are displayed as crosses. Weak CA, subject group who observed a weakly control-averse peer; No Peer, subject group who did not observe a peer; Strong CA, subject group who observed a strongly control-averse peer.

Next, we compared the baseline of control-averse behavior in the group No Peer with that of two experimental groups with peer influence, namely a group who received information about a weakly control-averse peer (group Weak CA, *n* = 29) and a group who received information about a strongly control-averse peer (group Strong CA, *n* = 30). To capture the baseline behavior, only the first two trials prior to any peer information were included in this analysis, that is one trial in the Controlled condition and the Free condition, respectively. Based on previous work^1,4,21^, control-averse behavior is defined as lower chosen levels when the subjects’ choice is restricted (in the Controlled condition) than when the subject can decide freely (in the Free condition). Therefore, we computed the individual baseline level of control-averse behavior as the difference of a subject’s chosen level in the first trial of the Free condition minus the subject’s chosen level in the first trial of the Controlled condition. An ANOVA confirmed that the level of control-averse behavior at baseline did not differ significantly between groups (*F*(2, 79) = 0.27, *p* = 0.77; Fig. 4). Pairwise comparisons using Wilcoxon rank sum tests also revealed no significant differences (all *p* > 0.1).

### Peer effects on control-averse behavior

To test whether the peer influence had an effect on the individual control-averse behavior, we analyzed the data across all three experimental groups, in which subjects either received information about the choices of a strongly control-averse peer (group Strong CA, *n* = 30), a weakly control-averse peer (group Weak CA, *n* = 29) or no peer information (group No Peer, *n* = 23). The peer information was presented after two thirds of the subjects’ own choices, randomly interspersed across the task. Specifically, subjects were asked to guess what a peer had chosen in the same situation, i.e. in the Controlled condition or the Free condition, respectively (Fig. 2B). This guessing component was included to provide a justification for the presentation of peer information and to ensure that subjects paid attention to the peer’s choice, which was otherwise irrelevant to the subjects’ payoff. When they were done guessing, subjects were presented with what the peer had chosen (Fig. 2C,D). Subjects were told that the peer was a student of a similar age who had participated in a previous session of the experiment, and that the peer remained identical throughout the task. In reality, the presented peer choices were selected by an algorithm programmed to mimic the behavior of a real subject from a pilot study (see Methods for details). Importantly, the peer information was always presented at the end of a trial and only in two thirds of the trials, whereas the Controlled and Free conditions were implemented in half of the trials each, in random order. Therefore, the peer influence occurs over the history of trials and should not be specific to the most recently observed peer choice.

We hypothesized that the type of peer information would moderate the effect of the choice restriction on the chosen level. Specifically, we hypothesized that the effect of the choice restriction on the chosen level would be greater in the group Strong CA (with information about a strongly control-averse peer) than in the other groups, and that it would be smaller in the group Weak CA (with information about a weakly control-averse peer) than in the other groups. To test these hypotheses, we set up a generalized linear mixed effects model (GLMM) with the chosen level in each trial as dependent variable. The predictors were Control_ij_, which indicated trials in the Controlled condition, and Strong CA_i_ and Weak CA_i_, which indicated subjects in the group Strong CA and subjects in the group Weak CA, respectively, using the group No Peer (without peer information) as a reference. The model further included a random-effects intercept for each subject as well as a random-effects slope for Control_ij_ within subjects.

**Figure 5:**
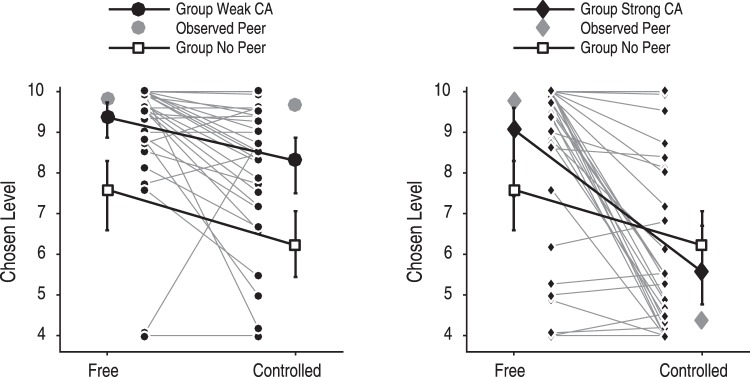
Peer effects on control-averse behavior. Shown here are the chosen levels in the Free and the Controlled condition for subjects in the group who observed a weakly control-averse peer (group Weak CA) and subjects in the group who observed a strongly control-averse peer (group Strong CA), respectively, compared with subjects in the group without peer information (group No Peer). For subjects in the groups with peer information, only trials after the first peer information are included in this figure, and for the subjects in the group without peer information only trials after the first two baseline trials are included in this figure. The error plots show the respective point estimators and 95% confidence intervals of the Hodge-Lehman medians. The gray symbols depict the means of the observed peer’s chosen levels, and the connected data points in the center show individual subjects’ means in the respective group with peer information.

We find that, in line with our hypothesis, the effect of the Controlled condition on the chosen level is moderated by peer influence (Table 1, Fig. 5): The decrease of the chosen level in the Controlled condition compared with the Free condition is significantly greater for subjects in the group Strong CA than for subjects in the group No Peer (*β* = −1.25, *t*(2946) = −2.39, *p* = 0.017, 95% CI [−2.28, −0.23]). By contrast, subjects in the group Weak CA show no significant interaction effect: The decrease of the chosen level in the Controlled condition compared with the Free condition is similar for subjects in the group Weak CA and for subjects in the group No Peer (*β* = 0.20, *t*(2946) = 0.37, *p* = 0.708, 95% CI [−0.84, 1.23]). In a direct comparison of the parameter estimates, subjects in the group Strong CA show a significantly more negative effect of the Controlled condition on the chosen level than subjects in the group Weak CA (linear combination of the respective coefficients: *β* = −1.45, *F*(1, 2946) = 8.68, *p* = 0.003). Moreover, we find that subjects in the group Weak CA chose higher levels compared with the group No Peer in the Free condition (*β* = 1.43, *t*(2946) = 2.81, *p* = 0.005, 95% CI [0.43, 2.43]) and in the Controlled condition (linear combination of the respective coefficients: *β* = 1.63, *F*(1, 2946) = 5.01, *p* = 0.025). Inspection of the subjects’ mean chosen levels suggests that this behavior closely resembles the observed peer choices (Fig. 5). Therefore, in both groups with peer information, subjects’ followed the observed peer behavior.

**Table 1:** Results of the GLMM testing the peer effects on control-averse behavior.

	*β* estimate	*SE*	*t*(2946)	*p*	95% CI	
					Lower	Upper
Control_ij_	−1.13	0.39	−2.87	0.004	−1.91	−0.36
Strong CA_i_	0.85	0.51	1.68	0.093	−0.14	1.84
Weak CA_i_	1.43	0.51	2.81	0.005	0.43	2.43
Control_ij_*Strong CA_i_	−1.25	0.52	−2.39	0.017	−2.28	−0.23
Control_ij_*Weak CA_i_	0.20	0.53	0.37	0.708	−0.84	1.23
(Intercept)	7.52	0.38	19.78	<0.001	6.77	8.26
**Random-effects intercept for subjects (82 levels)**						
Estimated *SD*	1.81					
**Random-effects slope for Control**_**ij**_ **within subjects (82 levels)**						
Estimated *SD*	1.86					
R^2^	0.79					
BIC	9497.40					

*Note*. The dependent variable is the chosen level by subject *i* in trial *j*. The predictor Control_ij_ is equal to 1 in the Controlled condition and 0 otherwise. Strong CA_i_ is equal to 1 for subjects in the group who observed a strongly control-averse peer and 0 otherwise, and Weak CA_i_ is equal to 1 for subjects in the group who observed a weakly control-averse peer and 0 otherwise. Subjects in the group without peer information served as reference group. The model includes a random-effects intercept for each subject and a random-effects slope for Control_ij_ within subjects. Sample size N = 82.

To check the robustness of our results with regard to censoring of the data, we ran an additional Bayesian hierarchical censored linear regression that controlled for the censoring of the data at the upper and lower end of the dependent variable (Supplementary Table S2), and a Bayesian hierarchical ordinal regression that treats the values of the dependent variable as distinct, ordered categories instead of a linear scale (Supplementary Table S3). Both additional models confirm the results of the GLMM: a significant main effect of Control, a significant interaction effect of Control * Strong CA, and a significant main effect of the group Weak CA, as indicated by credible intervals not crossing zero. Also in line with the GLMM, the interaction effect of Control * Weak CA and the main effect of Strong CA remain not significant, as indicated by credible intervals crossing zero. Together, these results support our hypothesis that peer influence can modulate control-averse behavior.

### Effects of the most recent peer information on control-averse behavior

To test whether the individual choice behavior is influenced by the peer’s most recent choice rather than the peer’s control-averse behavior, we ran an additional GLMM that included only data from groups with peer information, i.e. groups Strong CA and Weak CA (n = 59). The dependent variable was the chosen level in each trial. The predictors were Control_ij_, which indicated trials in the Controlled condition, and PeerChoice_ij-1,_ which represents the most recently presented chosen level by the peer for a given trial. Critically, due to the randomized trial sequences the most recent peer information could be either of the Controlled or the Free condition and did not necessarily match the condition of the current trial. This allowed us to test the effect of the peer’s prosocial behavior independent of the peer’s control-averse behavior. The model further included a random-effects intercept for each subject as well as random-effects slopes for Control_ij_ and for PeerChoice_ij-1_ within subjects.

This GLMM revealed that the most recent peer choice as well as its interaction with the Controlled condition has no significant effect on the chosen level (*p* > 0.1, Table 2), whereas the effect of the Controlled condition on the chosen level remains significant (*p* < 0.001). This suggests that the subject’s behavior is not simply influenced by the peer’s most recent prosocial behavior, but by the peer’s control-averse behavior, i.e. the peer’s behavior depending on the Controlled condition.

**Table 2:** Results of the GLMM testing the effects of the most recent peer information on control-averse behavior.

	*β* estimate	*SE*	*t*(1911)	*p*	95% CI	
					Lower	Upper
Control_ij_	−1.64	0.34	−4.78	<0.001	−2.32	−0.97
PeerChoice_ij-1_	0.02	0.02	1.30	0.194	−0.01	0.06
Control_ij_*PeerChoice_ij-1_	−0.01	0.02	−0.37	0.710	−0.06	0.04
(Intercept)	8.50	0.28	30.66	<0.001	7.95	9.04
**Random-effects intercept for subjects (59 levels)**						
Estimated *SD*	1.81					
**Random-effects slope for Control**_ij_ **within subjects (59 levels)**						
Estimated *SD*	2.10					
**Random-effects slope for PeerChoice**_**ij-1**_ **within subjects (59 levels)**						
Estimated *SD*	<0.01					
R^2^	0.82					
BIC	6094					

*Note*. The dependent variable is the chosen level by subject *i* in trial *j*. The predictor Control_ij_ is equal to 1 in the Controlled condition and 0 otherwise. PeerChoice_ij-1_ is the most recently presented chosen level by the peer. Only subjects in the groups with peer information were included in this analysis. The model includes a random-effects intercept for each subject and random-effects slopes for Control_ij_ and PeerChoice_ij-1_ within subjects. Sample size N = 59.

### Potential moderators of the peer effects on control-averse behavior

Next, we asked whether the effect of peer influence on control-averse behavior remains robust when we control for a moderation by subjects’ general resistance to peer influence as measured independently from the task using the Resistance to Peer Influence (RPI) scale (Supplementary Information S1)^17^. The RPI scores do not differ between the three groups as assessed by an ANOVA (*F*(2,79) = 1.70, *p* = 0.190). Pairwise comparisons reveal that subjects in the group Strong CA (*M* = 2.91 ± 0.35 *SD*, *Mdn* = 2.90) have slightly lower RPI scores than subjects in the group Weak CA (*M* = 3.06 ± 0.28 *SD*, *Mdn* = 3.10; Wilcoxon rank sum test, *z* = −1.97, *p*_*uncorrected*_ = 0.048, effect size *r* = −0.26). However, this difference is not significant after correction for multiple comparisons. To control for a potential moderation of the peer effect, we added a main effect of and interactions with RPI_i_ to the GLMM, where RPI_i_ is the normalized and mean-centered score of the general resistance to peer influence as measured by the RPI scale. The GLMM reveals no significant three-way interaction and therefore no moderation of the peer effects on control-averse behavior by the RPI score (*p* > 0.1; Supplementary Table S4), whereas the peer effect on control-averse behavior remains significant (*p* = 0.011).

Finally, we investigated whether the effect of peer influence on control-averse behavior remains robust when we control for a moderation by the subjects’ general tendency to rebel against restrictions of their freedom of choice as measured by the Hong Psychological Reactance Scale (HPRS)^19,20^. The HPRS scores do not differ between groups as assessed by an ANOVA (*F*(2,79) = 0.56, *p* = 0.573). Pairwise comparisons using Wilcoxon rank sum tests also reveal no significant differences (all *p* > 0.1). To control for a moderation of the peer effect by the HPRS score, we ran an additional GLMM. This GLMM was identical to the first GLMM, except that we added a main effect of and interactions with HPRS_i_, which is the normalized and mean-centered HPRS score. This GLMM reveals no significant three-way interaction and therefore no moderation of the peer effects on control-averse behavior by the HPRS score (Supplementary Table S5), whereas the peer effect on control-averse behavior remains significant (*p* = 0.012). When testing the individual HPRS subscales in separate GLMMs, we also find no significant moderation effect (all *p* > 0.1; Supplementary Table S6).

## Discussion

Peer influence on social behavior has rarely been studied in controlled experiments with real consequences. In particular, it has remained unknown whether peer influence affects a psychologically and clinically highly relevant phenomenon, control-averse behavior. Control-averse behavior describes the negative response to exogenous control of one’s decisions and can impede important social interactions, for example between therapists and patients, or employers and employees. It is therefore important to identify factors that might amplify or attenuate control-averse behavior. Using a novel experimental paradigm with real monetary consequences, we found that control-averse behavior could indeed be influenced by peer behavior. Specifically, individuals who were informed about the choices of a strongly control-averse peer displayed increased control-averse behavior compared with individuals who remained uninformed or individuals who were informed about the choices of a weakly control-averse peer. Moreover, individuals followed the peer behavior even when this resulted in lower profits for themselves. Hence, we demonstrate peer effects on both costly and control-averse social behavior.

A critical feature of our study was that subjects had no systematic incentives to follow the peer, because their own profit was independent from the peer’s profit. Nonetheless, subjects adopted the peer’s behavior both when the peer was more generous, thereby accepting a lower own profit, and when the peer was more control-averse, thereby selecting less generous and more selfish options. If subjects simply used the peer information to justify more selfish choices, we should see a peer effect only when the peer chose a low level, so only for the group with information about a strongly control-averse peer in the Controlled condition. However, we also find a peer effect when the peer chose a high level, i.e. in the Free condition and for subjects in the group with information about a weakly control-averse peer. Therefore, subjects followed the peer even when choices were costly. In this regard, our findings contradict the assumption of classic social preference models according to which behavior should not be influenced by peer behavior that is unrelated to the own costs and benefits, as well as a recent finding that peer influence has negative, but not positive effects on social behavior^13^. Our findings, in contrast, reveal both positive and negative effects of peer influence. They thereby extend previous findings of positive effects of peer influence^7,10^ and spillover effects from group environments to private prosocial choices^22^.

The effects of peer influence have often been attributed to conformity, suggesting that individuals tend to express the same behavior as a group^23^. Each subject in our study, however, was only confronted with a single peer’s behavior instead of a groups’ behavior. Likewise, given the absence of an audience and the use of an incentive scheme designed to motivate subjects to behave according to their true preferences, public or superficial conformity seem to be insufficient explanations for the peer effects we observe^23^. Subjects were also informed that the peer herself or himself had not received any information about other people’s behavior. It therefore seems unlikely that subjects might have had the impression that the peer was better informed and displayed the more advantageous behavior^24^. We can further dismiss the possibility that subjects feared a bad reputation^25^, because all decisions were anonymous. Furthermore, in contrast to previous studies that have investigated the effects of peer feedback on prosocial decisions^11,16^, the peer influence in our study did not involve any type of social evaluation.

An alternative explanation is that the subjects might have inferred a social norm from the peer’s behavior, although it was only a single person. Previous work has suggested that a single peer’s behavior may function as a reminder of a social norm or a heuristic for their own behavior^26,27^. In line with this reasoning, the generous and weakly control-averse peer might have reminded the subjects of the norm to be generous and fair, whereas the strongly control-averse peer might have reminded the subjects of the norm to punish the player A’s distrustful decision to control. Other work discusses that the information about a peer’s choice might be used to infer the quality of a choice option^28^. In other words, an option may become more attractive simply because other people have chosen it. Likewise, our subjects might have inferred that the levels chosen by the peer have a greater utility, on top of or despite the associated profits.

Another possibility is that subjects changed their actual preferences. Such an internalization of the peer’s preferences may have occurred as a by-product of learning about the peer’s preferences^29^. Although subjects had no systematic monetary incentive to follow the peer, guessing the peer’s choice correctly may have been rewarding in itself and thereby may have reinforced adopting the peer’s behavior. In other words, simply acquiring information about the peer’s preferences may have changed the subjects’ own preferences—in many cases even if that preference was associated with lower payoffs for the subjects. This would be in accordance with a recent finding of peer effects on intentions to volunteer that lasted well into a private setting, during which subjects had no incentive to conform with the peer group^6^. Whereas their study showed effects of a peer group on self-reported intentions, our study shows effects of a single peer on actual (not hypothetical) social decisions.

Finally, a recent study has proposed that individuals might follow a deviant peer’s behavior as a form of restoring their freedom of choice^18^. The authors found that individuals with higher HPRS scores were more likely to comply with a peer’s request to engage in deviant behavior, such as drinking. The authors interpret this compliance with peer influence as the subjects’ way of restoring their autonomy to choose a deviant behavior. Critically, the chosen deviant behavior in that study remained hypothetical and was not actually implemented. In line with Leander *et al*., we find that subjects in the group with a strongly control-averse peer comply with the peer’s less generous and somewhat deviant choice in the Controlled condition, which could be a way to demonstrate autonomy in response to the choice restriction. However, subjects in both groups with peer information also followed the more generous peer behavior in the Free condition, in which case compliance with the peer behavior cannot be interpreted as deviant behavior. Moreover, unlike Leander *et al*. we did not find a significant association between the HPRS score and the compliance with the peer influence. Therefore, we cannot conclude that the peer effect we observe is an attempt to pursue autonomy.

The current study may also expand the scope of current neuroscientific research on peer influence, which has overwhelmingly focused on the effects of peer groups. Neuroimaging studies, for example, have found that the information about a peer group’s attitudes^30^ or the mere presence of a peer group^16^ had an effect on behavior and correlated with activation changes in brain regions associated with social decision making, suggesting that similar neural networks might be involved for social behavior and sensitivity to peer influence. Here, we demonstrate the effects of a single peer, rather than a peer group, and introduce an experimental paradigm that allows researchers to measure the individual susceptibility to peer influence on control-averse behavior. Building on previous work on the neural basis of control-averse behavior^4,5^, this study prepares the ground for fruitful future investigations of the neural processes underlying the peer effects on control-averse behavior.

In conclusion, our results indicate that even information about a single, anonymous peer can contribute to the complex phenomenon of control-averse behavior. In particular, information about a strongly control-averse peer can contribute to an increase of individual control-averse behavior. This is relevant for many domains of our society, in which successful social interactions rely on the interaction partner’s compliance, such as therapist-patient interactions, parent-child interactions or employer-employee interactions. Furthermore, the fact that our subjects’ behavior had real consequences for themselves as well as a third person speaks to the generalizability of our findings to social interactions outside of the laboratory, which has important implications for clinical work with patients. Our study suggests that to destabilize compliance, all it takes is information about one non-compliant peer. On the bright side, we also find that information about a single generous peer can lead to an increase of generous behavior. Future studies could build on these findings to develop and test applications in the field.

## Methods

### Participants

We recruited a total of 84 students from the University of Bern for participation in this study. The sample size was determined by a statistical power analysis as implemented in G*Power^31^. Based on the effect size r = 0.56 of control-averse behavior in a previous data set^4^, the power analysis suggested that to achieve this effect size at *p* < 0.05 with a power of approximately 0.80 in a one-tailed Wilcoxon signed rank test, a sample size of 23 participants would be required. Hence, we recruited 23 to 30 participants for each of the three experimental groups described in the next section.

Students of economics, psychology and social sciences were excluded from participation to reduce the possibility of prior knowledge of the concept of control aversion or social influence theory. Further exclusion criteria were left-handedness, smoking and a reported history of psychological disorders, neurological or cardiovascular diseases, because participants also underwent magnetic resonance imaging while engaging in the task described in the next section. The imaging analyses are beyond the scope of this paper and will be reported elsewhere. One participant was excluded due to technical problems during data acquisition and another participant due to a neurological disease discovered after data acquisition. The remaining 82 participants were included in the analysis. All experimental protocols were approved by the Bern Cantonal Ethics Commission. The methods were carried out in accordance with the relevant guidelines and regulations. All participants gave informed, written consent and received a compensation of CHF 50 (≈USD 50) for participation in the study in addition to the payoff from the task described next.

### Control aversion task with and without peer influence

Subjects were randomly assigned to one of three groups, in which they received information about a strongly control-averse peer (group Strong CA, *n* = 30), information about a weakly control-averse peer (group Weak CA, *n* = 29) or no peer information (group No Peer, *n* = 23). Experimenters were blind as to whether a subject was assigned to the group Strong CA or Weak CA and the task instructions were identical in these two groups. Each group completed a different version of the Control aversion task, which will be described in detail below. In short, an individual called player A either lets the subject make a free choice or restricts the subject’s choice options and thereby exerts control over the decision. The subject, labeled player B, then chooses a monetary allocation that will affect both their own and the player A’s payoff. On a subset of trials, peer information is presented as the allocation chosen by an individual called player B*, a student of the same age range who had faced the same decisions as the subject.

In total, subjects were presented with 36 anonymous players A’s decisions from a pilot study. Subjects were informed that the players A’s decisions had been prerecorded for logistic reasons, but that their choices in the task had real consequences in the sense that one trial would be randomly selected and paid out to themselves and the corresponding player A. Subjects in the groups with peer information were further told that they would see choices made by a peer, labeled player B*, a student between 18–35 years who had responded to our invitation email—just like themselves—and participated in a previous session of the experiment. In reality, the peer’s choices were programmed with simple algorithms that mimicked the behavior of strongly or weakly control-averse participants from a pilot study, respectively. This was done to ensure that subjects would see one of two extremes of a peer’s control-averse behavior and to ensure homogeneity of the peer influence within each group with peer information. Care was taken that the peer behavior reflected realistic choices that had actually occurred in our pilot study. Concretely, none of our subjects in the pilot study had made highly selfish choices when they could decide freely or had been more generous when their choice options were restricted than when they could decide freely. Neither did any subject believe that other subjects would behave this way. Therefore, to ensure the credibility of the peer’s existence these peer behaviors were not included in the experiment. No subject voiced any doubts about the existence of the peer in a post experimental debriefing. Prior to performing the task, subjects read the instructions and were quizzed to ensure they had understood the task and its payoff scheme.

We now describe the task and its different versions in detail. All subjects made repeated monetary allocation decisions in two conditions as follows (Fig. 2). In the Free condition, subjects had the choice between ten monetary allocations, called generosity levels one to ten (from left to right). In the Controlled condition, player A requested a minimum of level four and thereby restricted the subjects’ choice to levels four to ten. The generosity levels ranged from a selfish allocation (98 points for the subject, 9 points for the player A) to a more generous and fair allocation (80 points for both the subject and the player A). With increasing generosity levels, the player A’s profit increased linearly (in increments of seven or eight points) whereas the subjects’ profit decreased linearly (in increments of two points). The monetary allocations were specifically designed to motivate subjects to choose a high level in the Free condition and to create room for the choice of a lower level in the Controlled condition, which is a prerequisite for measuring control-averse behavior^21^. To achieve this, we built on subjects’ preference for equality and efficiency^32^ and designed the levels such that the highest level represents both an equal allocation between player A and B as well as the largest sum of points. We visualized the levels as stacked color bars, such that each player was assigned one specific color throughout the task: For example, player A’s profit was represented by orange bars, the subject’s profit by blue bars and—when peer information was presented—the peer’s (player B*’s) profit was represented by green bars. The colors assigned to each player were counterbalanced across subjects. The subjects’ (or the peer’s) and player A’s points were also printed below and above the color bars, respectively. This way subjects always received the exact information about the points, but were also given an intuitive, easy-to-grasp visualization of the point allocations in each level. Subjects selected a generosity level by moving a selection frame that appeared on a random (available) level to the desired level and selecting OK via button presses.

The players A’s decisions were preselected such that the subjects completed the same number of trials in the Free and in the Controlled condition, i.e. 18 trials per condition. In the first two trials of the task, all subjects completed one trial in the Controlled condition and one trial in the Free condition without peer information, in random order. Based on previous work^1,4,21^, control-averse behavior is defined as lower chosen levels when player A requests a minimum level (in the Controlled condition) than when the subject can decide freely (in the Free condition). Therefore, the difference between the chosen level in the first trial of the Free condition minus the chosen level in the first trial of the Controlled condition serves as a baseline measure of the individual level of control-averse behavior. The remaining trials were presented in random order.

For the subjects in the group without peer information (group No Peer), all 36 trials ended after the choice of a generosity level (Figs 1 and 2A). For the subjects in the groups with peer information, 12 randomly interspersed trials (33%) also ended there (trials without peer information); in the remaining 24 trials (66%), subjects were asked to guess what the peer (player B*) had chosen in the same situation (Figs 1 and 2B). Then they were presented with the peer’s choice (trials with peer information, Fig. 2C,D). Note that peer information was always presented at the end of a trial and only in two thirds of the trials, whereas the Controlled and Free conditions were implemented in half of the trials each, in random order. This leads to randomized trial sequences, in which a subject might see, for example, a peer’s choice in the Controlled condition, but subsequently will be asked to make a choice in the Free condition. This design feature helps to disentangle the direct effect of the most recent peer choice (e.g. a selfish choice) from the more indirect effect of the peer’s control-averse behavior that a subject has observed over the history of trials (e.g. selfish choices in the Controlled condition, but generous choices in the Free condition). Importantly, subjects were instructed that player B* remained identical throughout the experiment and that we were interested in how well they could predict player B*’s choices. This feature was included to motivate subjects to pay attention to the peer’s choices as well as a justification for the presentation of peer information. In the Free condition, both groups with peer information observed the choices of a peer who chose level ten with a likelihood of 80% and level nine with a likelihood of 20%. In the Controlled condition, the group Strong CA observed the choices of a peer who chose level four with a likelihood of 70% and level five with a likelihood of 30%, reflecting strongly control-averse behavior. By contrast, the group Weak CA observed the choices of a peer who chose level ten with a likelihood of 70% and level nine with a likelihood of 30%, reflecting more generous and weakly control-averse behavior. The variability in the peer’s choices was implemented for verisimilitude and to keep subjects engaged in guessing the peer’s choices. Critically, the peer’s choices were independent of the subjects’ payoffs.

At the end of the task, one trial was randomly selected for payoff to the subject and the matched player A. The profits in the selected trial were converted into CHF (with 1 point = CHF 0.20 ≈ USD 0.20). Based on the task, the subjects’ received a mean CHF 17.00 ± 1.10 *SD*, and the players A received a mean CHF 12.00 ± 4.40 *SD*.

### Analysis of peer effects on control-averse behavior

To test the effects of peer influence on control-averse behavior, we set up a GLMM using the function fitglme as implemented in the MATLAB Statistics and Machine Learning Toolbox (R2015b, MathWorks). The dependent variable of this GLMM was the chosen level by subject *i* in trial *j*. The predictors modeled whether the subject’s choice was restricted (Controlled condition) or free (Free condition) for a given trial, and what type of peer information each subject received (strongly control-averse, weakly control-averse, or no peer information). Note that the statistical test was corrected for a bottom effect between the conditions, following the procedure by Falk and Kosfeld^1^: chosen levels in the Free condition that were smaller than level four were set to level four, which is the smallest possible level in the Controlled condition. The GLMM included a predictor Control_ij_ that is equal to 1 for trials in the Controlled condition and 0 otherwise. The groups with peer information were included as categorical predictors Strong CA_i_ and Weak CA_i_, using the group No Peer as reference. Strong CA_i_ is equal to 1 for subjects in the group with information about a strongly control-averse peer and 0 otherwise, and Weak CA_i_ is equal to 1 for subjects in the group with information about a weakly control-averse peer and 0 otherwise. We further included random-effects intercepts for subjects and random slopes for Control_ij_ within subjects. Visual inspection of residual plots did not reveal any obvious deviations from homoscedasticity or normality.

To check the robustness of the GLMM results with regard to censoring of the data, we implemented two additional models. First, we ran a Bayesian hierarchical censored linear regression that controls for the censoring of the data at the upper and lower end of the dependent variable. Second, we ran a Bayesian hierarchical ordinal regression that treats the dependent variable as distinct, ordered categories. Both additional regression models were estimated using Bayesian Markov-chain Monte Carlo methods with R (version 3.2.4)^33^ in combination with STAN^34^, using the default, uninformative priors specified by the R-package brms (version 1.10.2)^35^.

### Analysis of the effects of the most recent peer information on control-averse behavior

To test whether the subjects’ behavior was influenced by the most recently presented peer information in addition to the Controlled condition, we ran a new GLMM using the data from the two groups with peer information, group Strong CA and group Weak CA (n = 59). The dependent variable of this GLMM was the chosen level by subject *i* in trial *j*. The predictors modeled whether the subject’s choice was restricted (Controlled condition) or free (Free condition) for a given trial, and the most recently observed choice of the peer. A predictor for the type of peer information (Strong CA, Weak CA) was omitted from the model due to rank deficiency of the design matrix.

More specifically, the GLMM included a predictor Control_ij_ that is equal to 1 for trials in the Controlled condition and 0 otherwise, and a predictor PeerChoice_ij-1_, which was the most recently presented level chosen by the peer (see Fig. 2C,D). Note that the first trials before any peer information were dropped. Moreover, due to the randomized trial sequences the most recent peer information could be either of the Controlled or the Free condition and therefore did not necessarily match the condition of the current trial. Furthermore, because peer information was presented in only two thirds of the trials, the most recent peer information could be more than one trial ago. For trials between peer information, missing values for the predictor PeerChoice_ij-1_ were filled with the last available peer information for the respective subject. We further included random-effects intercepts for subjects and random slopes for Control_ij_ and for PeerChoice_ij-1_ within subjects.

### Analysis of potential moderators of the peer effects

To investigate whether the peer effects on control-averse behavior might be moderated by the subjects’ general resistance to peer influence, we asked subjects to fill in a German version of the Resistance to Peer Influence (RPI) scale (for the German version see Supplementary Information S1)^17^. To maintain linguistic validity the RPI scale was translated into German by the first author of the current study, then back-translated into English by a professional translator and compared with the original. In the RPI scale, subjects are asked to indicate which of ten pairs of statements best describes them as a person, for example: “Some people go along with their friends just to keep their friends happy” BUT “Other people refuse to go along with what their friends want to do, even though they know it will make their friends unhappy”. Responses are coded on a 4-point Likert scale, ranging from 1 = *really true* and 2 = *sort of true* for one statement to 3 = *sort of true* and 4 = *really true* for the other statement. In our sample, the RPI scale had an excellent internal consistency (Cronbach’s *α*= 0.96). The overall score of general resistance to peer influence (‘RPI score’) was computed as described in the Supplementary Information S1. On average, subjects had an RPI score of mean 2.97 ± 0.33 *SD* (*Mdn = *2.95, range: 2.2–4).

To control for the RPI score as a potential moderator of the peer effects, we ran a new GLMM, which was identical to the first GLMM, except that we added the moderator variable RPI_i_, which is the normalized and mean-centered score of the general resistance to peer influence as measured by the RPI scale. Specifically, we added a predictor RPI_i_, its interactions with Strong CA_i_ and Weak CA_i_, and three-way interactions of Control_ij_, Strong CA_i_ and Weak CA_i_, respectively, and RPI_i_ to the GLMM. Because we did not assume that the resistance to peer influence should affect control-averse behavior in the absence of peer influence, we omitted the interaction of RPI_i_ and Control_ij_ from the GLMM.

To account for the possibility that the peer effects on control-averse behavior might be moderated by subjects’ general tendency to rebel against restrictions of their freedom of choice, we asked subjects to fill in a German version of the Hong Psychological Reactance Scale (HPRS)^19,20,36^. The HPRS consists of 14 items that describe general attitudes and habits. Subjects rated these items on a 5-point Likert scale ranging from 1 = *strongly disagree* to 5 = *strongly agree*. Based on the subjects’ ratings, we computed the scores of the four subscales Emotional response toward restricted choices, Reactance to compliance, Resisting influence from others, Reactance toward advice and recommendations. All subscales of the HPRS had good to excellent internal consistencies (Cronbach’s *α* between 0.85 and 0.99). To achieve one overall score of the HPRS (‘HPRS score’), we computed the mean of the four subscale scores for each subject. On average, subjects had an HPRS score of mean 3.04 ± 0.47 *SD* (*Mdn* = 3.14, range: 1.86–4.07).

To control for the HPRS score as a potential moderator of the peer effects, we ran a third GLMM, which was identical to the first GLMM, except that we added the moderator variable HPRS_i_, which is the normalized and mean-centered score of the general tendency to rebel against restrictions of one’s freedom of choice as measured by the HPRS. Specifically, we added a predictor HPRS_i_, its interaction with Control_ij_ and three-way interactions of Control_ij_, Strong CA_i_ and Weak CA_i_, respectively, and HPRS_i_ to the first GLMM. Because we assumed that the general tendency to rebel against restrictions of one’s freedom of choice is relevant for the effect of the Controlled condition, but not for the effect of the peer influence per se, we omitted the interactions of HPRS_i_ with Strong CA_i_ and Weak CA_i,_ respectively, from the GLMM.

## Supplementary information

Supplementary Information

## Data Availability

The data that support the findings of this study are available from the corresponding author S.R. upon request.
