# Supplementary material for: Peer effects on control-averse behavior
Source: Sci Rep. 2019 Feb 28;9:3012. doi: 10.1038/s41598-019-39600-9 (PMC6395657; doi:10.1038/s41598-019-39600-9)
Supplement: Supplementary file 1 — Supplementary Information [file 41598_2019_39600_MOESM1_ESM.pdf]

Supplementary Information

Peer effects on control-averse behavior

Sarah Rudorf[1*], Thomas Baumgartner[1], and Daria Knoch[1*]

[1]Department of Social Psychology and Social Neuroscience, Institute of Psychology, University of Bern, 3012 Bern, Switzerland.

*Correspondence should be addressed to:

sarah.rudorf@psy.unibe.ch or daria.knoch@psy.unibe.ch

S 1. German version of the Resistance to Peer Influence scale (RPI scale, Steinberg & Monahan, 2007).

Entscheiden Sie bitte für jede der folgenden Fragen, welche Art von Person Sie am ehesten sind – die Person, die auf der linken Seite beschrieben ist, oder die Person, die auf der rechten Seite beschrieben ist. Dann entscheiden Sie, ob die Beschreibung für Sie „eher zutrifft“ oder „genau zutrifft“ und kreuzen das entsprechende Kästchen an. Markieren Sie für jede Zeile bitte genau EINS der vier Kästchen.

Trifft für mich **genau zu**	Trifft für mich **eher zu**				Trifft für mich **eher zu**	Trifft für mich **genau zu**
☐	☐	Manche Leute ziehen mit ihren Freunden mit, nur um ihre Freunde bei Laune zu halten.	ABER	Andere Leute weigern sich, mit ihren Freunden mitzuziehen, auch wenn sie wissen, dass es ihre Freunde unglücklich macht.	☐	☐
☐	☐	Manche Leute finden es wichtiger individuell zu sein als sich der Menge anzupassen.	ABER	Andere Leute finden es wichtiger, sich der Menge anzupassen statt als Individuum herauszustechen.	☐	☐
☐	☐	Bei manchen Leuten ist es so, dass ihre Freunde sie ziemlich leicht dazu kriegen, ihre Meinung zu ändern.	ABER	Bei anderen Leuten ist es so, dass ihre Freunde sie nur ziemlich schwer dazu kriegen, ihre Meinung zu ändern.	☐	☐
☐	☐	Manche Leute würden etwas tun, von dem sie wissen, dass es falsch ist, nur um die Zustimmung ihrer Freunde zu erhalten.	ABER	Andere Leute würden etwas, von dem sie wissen, dass es falsch ist, nicht tun, nur um die Zustimmung ihrer Freunde zu erhalten.	☐	☐
☐	☐	Manche Leute verstecken ihre wahre Meinung vor ihren Freunden, wenn sie denken, ihre Freunde werden sich deshalb über sie lustig machen.	ABER	Andere Leute sagen ihre wahre Meinung vor ihren Freunden, auch wenn sie wissen, ihre Freunde werden sich deshalb über sie lustig machen.	☐	☐
☐	☐	Manche Leute würden das Gesetz nicht brechen, nur weil ihre Freunde sagen, sie würden es tun.	ABER	Andere Leute würden das Gesetz brechen, wenn ihre Freunde sagen, sie würden es brechen.	☐	☐
☐	☐	Manche Leute verhalten sich so anders, wenn sie mit ihren Freunden zusammen sind, dass sie sich fragen, wer sie „wirklich sind“.	ABER	Andere Leute verhalten sich genauso wenn sie alleine sind wie wenn sie mit ihren Freunden zusammen sind.	☐	☐
☐	☐	Manche Leute gehen mehr Risiken ein, wenn sie mit ihren Freunden zusammen sind als wenn sie alleine sind.	ABER	Andere Leute verhalten sich genauso risikofreudig, wenn sie alleine sind wie wenn sie mit ihren Freunden zusammen sind.	☐	☐
☐	☐	Manche Leute sagen Dinge, die sie gar nicht wirklich meinen, denn sie glauben, ihre Freunde respektieren sie dann mehr.	ABER	Andere Leute würden Dinge, die sie nicht wirklich meinen, nicht sagen, nur um ihre Freunde dazu zu kriegen, sie mehr zu respektieren.	☐	☐
☐	☐	Manche Leute glauben, es ist besser ein Individuum zu sein, selbst wenn Andere es ärgert, dass man gegen den Strom schwimmt.	ABER	Andere Leute glauben, es ist besser mitzumachen als Andere zu verärgern.	☐	☐

Note. Translated into German by SR. To maintain linguistic validity, the translation was back-translated by a professional translator and compared with the original. Each item (i.e. each line) is scored from 1 to 4,

reading from left to right. The scores for items 2, 6, and 10 need to be reversed. To compute the overall RPI score, divide the sum of scores for all valid responses by the number of valid items. Steinberg and Monahan (2007) recommend that at least 7 items have valid responses.

S 2. Results of the Bayesian hierarchical censored linear regression model testing the peer effects on control-averse behavior.

Dependent variable:	Chosen $Level_{ij}$			
			95% credible interval	
	Mean estimate	SD	Lower	Upper
$Control_{ij}$	−0.18	0.08	−0.33	−0.03
Strong CA_i	0.17	0.12	−0.06	0.39
Weak CA_i	0.26	0.12	0.03	0.49
$Control_{ij}$*Strong CA_i	−0.27	0.10	−0.46	−0.06
$Control_{ij}$*Weak CA_i	−0.05	0.10	−0.25	0.16
(Intercept)	0.81	0.09	0.65	0.99
Random-effects intercept for subjects (82 levels)				
Estimated *SD*	0.41			
Random-effects slope for Control within subjects (82 levels)				
Estimated *SD*	0.34			
R^2	0.73			
Posterior samples	3000			
Observations	2952			

Note. The dependent variable is the chosen level by subject *i* in trial *j*, which is normalized to the interval (0, 1). The predictor $Control_{ij}$ is equal to 1 in the Controlled condition and 0 otherwise. Strong CA_i is equal to 1 for subjects in the group who observed a strongly control-averse peer and 0 otherwise, and Weak CA_i is equal to 1 for subjects in the group who observed a weakly control-averse peer and 0 otherwise. Subjects in the group without peer information served as reference group. The model includes indicators specifying censored values at the upper and lower ends of the dependent variable, a random-effects intercept for each subject and a random-effects slope for $Control_{ij}$ within subjects. It was estimated using Bayesian Markov-chain Monte Carlo methods, using uninformative priors. Model fits are given as the population level mean of the posterior distribution ± standard deviation (SD) and the 95% credible

interval. Sample size N = 82. Weak CA, subject group who observed a weakly control-averse peer; Strong CA, subject group who observed a strongly control-averse peer.

S 3. Results of the Bayesian hierarchical ordinal regression model testing the peer effects on control-averse behavior.

Dependent variable:	Chosen $Level_{ij}$			
			95% credible interval	
	Mean estimate	SD	Lower	Upper
$Control_{ij}$	−2.13	0.82	−3.77	−0.45
Strong CA_i	1.83	1.31	−0.72	4.53
Weak CA_i	3.18	1.33	0.52	5.64
$Control_{ij}$*Strong CA_i	−3.00	1.12	−5.32	−0.87
$Control_{ij}$*Weak CA_i	−0.76	1.14	−2.98	1.49
(Intercept 1)	−4.46	0.98	−6.40	−2.59
(Intercept 2)	−3.18	0.98	−5.12	−1.32
(Intercept 3)	−2.18	0.98	−4.11	−0.28
(Intercept 4)	−1.11	0.98	−3.04	0.76
(Intercept 5)	0.28	0.98	−1.66	2.17
(Intercept 6)	2.13	0.98	0.23	4.00
Random-effects intercept for subjects (82 levels)				
Estimated SD	4.62			
Random-effects slope for Control within subjects (82 levels)				
Estimated SD	3.85			
Posterior samples	3000			
Observations	2952			

Note. The dependent variable is the chosen level by subject *i* in trial *j,* which is modeled as seven distinct, ordered categories (level four to level ten). The predictors are as described in S 2. The model includes a random-effects intercept for each subject and a random-effects slope for $Control_{ij}$ within subjects. It was estimated using Bayesian Markov-chain Monte Carlo methods, using uninformative priors. Model fits are given as the population level mean of the posterior distribution ± standard deviation (SD) and the 95%

credible interval. Sample size N = 82. Weak CA, subject group who observed a weakly control-averse peer; Strong CA, subject group who observed a strongly control-averse peer.

S 4. Results of the GLMM controlling for the moderation of the peer effects on control-averse behavior by the individual general resistance to peer influence.

					95% CI	
	β estimate	*SE*	*t*(2941)	*p*	Lower	Upper
$Control_{ij}$	−1.13	0.39	−2.92	0.004	−1.89	−0.37
Strong CA_i	0.79	0.50	1.57	0.116	−0.20	1.78
Weak CA_i	1.29	0.52	2.50	0.012	0.28	2.30
RPI_i	0.69	1.95	0.36	0.723	−3.14	4.53
$Control_{ij}$*Strong CA_i	−1.32	0.52	−2.54	0.011	−2.34	−0.30
$Control_{ij}$*Weak CA_i	0.34	0.53	0.64	0.524	−0.70	1.38
Strong CA_i*RPI_i	−2.08	2.60	−0.80	0.423	−7.17	3.01
Weak CA_i*RPI_i	1.77	2.96	0.60	0.550	−4.03	7.57
$Control_{ij}$* Strong CA_i*RPI_i	−2.07	1.77	−1.17	0.242	−5.53	1.40
$Control_{ij}$*Weak CA_i*RPI_i	−2.84	2.29	−1.24	0.216	−7.34	1.66
(Intercept)	7.53	0.38	19.94	< 0.001	6.79	8.27
Random-effects intercept for subjects (82 levels)						
Estimated *SD*	1.78					
Random-effects slope for Control within subjects (82 levels)						
Estimated *SD*	1.83					
R^2	0.79					
BIC	9532.60					

Note. The dependent variable is the chosen level by subject *i* in trial *j*. RPI_i is the normalized and mean-centered score of the RPI scale. The remaining predictors are as described in S 2. The model includes a random-effects intercept for each subject and a random-effects slope for $Control_{ij}$ within subjects. The subject group without any peer information served as reference group. Sample size N = 82 subjects. Weak CA, subject group who observed a weakly control-averse peer; Strong CA, subject group who observed a strongly control-averse peer.

S 5. Results of the GLMM controlling for the moderation of the peer effects on control-averse behavior by the general urge to restore one's freedom of choice as measured by the Hong Psychological Reactance Scale (HPRS, Hong & Faedda, 1996; Hong & Page, 1989).

					95% CI	
	β estimate	*SE*	*t*(2942)	*p*	Lower	Upper
$Control_{ij}$	−1.15	0.39	−2.97	0.003	−1.91	−0.39
Strong CA_i	0.83	0.51	1.64	0.101	−0.16	1.82
Weak CA_i	1.44	0.51	2.83	0.005	0.44	2.44
$HPRS_i$	−0.56	0.96	−0.58	0.561	−2.45	1.33
$Control_{ij}$*Strong CA_i	−1.29	0.52	−2.50	0.012	−2.30	−0.28
$Control_{ij}$*Weak CA_i	0.26	0.52	0.49	0.623	−0.76	1.27
$Control_{ij}$*$HPRS_i$	2.34	1.76	1.32	0.185	−1.12	5.80
$Control_{ij}$*Strong CA_i*$HPRS_i$	−4.00	2.35	−1.70	0.089	−8.61	0.61
$Control_{ij}$*Weak CA_i*$HPRS_i$	−3.89	2.53	−1.54	0.125	−8.86	1.08
(Intercept)	7.52	0.38	19.83	< 0.001	6.78	8.26
Random-effects intercept for subjects (82 levels)						
Estimated *SD*	1.80					
Random-effects slope for Control within subjects (82 levels)						
Estimated *SD*	1.82					
R^2	0.79					
BIC	9525.50					

Note. The dependent variable is the chosen level by subject *i* in trial *j*. $HPRS_i$ is the subject-wise mean score of the HPRS subscales, normalized and mean-centered across subjects. The remaining predictors are as described in S 2. The model includes a random-effects intercept for each subject and a random-effects slope for $Control_{ij}$ within subjects. The subject group without any peer information served as reference group. Sample size N = 82 subjects. Weak CA, subject group who observed a weakly control-averse peer; Strong CA, subject group who observed a strongly control-averse peer.

S 6. Results of the GLMMs controlling for a moderation of the peer effects on control-averse behavior by the subscales of the HPRS.

	Model 1		Model 2		Model 3		Model 4	
	β estimate	*SE*	*β* estimate	*SE*	*β* estimate	*SE*	*β* estimate	*SE*
$Control_{ij}$	−1.09**	0.39	−1.17**	0.39	−1.16**	0.40	−1.13**	0.39
Strong CA_i	0.86	0.50	0.82	0.50	0.82	0.51	0.85	0.50
Weak CA_i	1.47**	0.51	1.43**	0.51	1.43**	0.51	1.42**	0.51
$Control_{ij}$*Strong CA_i	−1.30*	0.52	−1.28*	0.52	−1.28*	0.53	−1.26*	0.52
$Control_{ij}$*Weak CA_i	0.22	0.52	0.23	0.52	0.24	0.53	0.22	0.52
$Emotion_i$	−0.92	0.90						
$Control_{ij}$*$Emotion_i$	1.83	1.75						
$Control_{ij}$*Strong CA_i*$Emotion_i$	−2.83	2.23						
$Control_{ij}$*Weak CA_i*$Emotion_i$	−4.08	2.46						
$Compliance_i$			−0.60	0.78				
$Control_{ij}$*$Compliance_i$			1.87	1.35				
$Control_{ij}$*Strong CA_i*$Compliance_i$			−3.61	1.95				
$Control_{ij}$*Weak CA_i*$Compliance_i$			−1.60	1.91				
$Influence_i$					−0.46	0.94		
$Control_{ij}$*$Influence_i$					0.93	1.74		
$Control_{ij}$*Strong CA_i*$Influence_i$					−2.18	2.30		
$Control_{ij}$*Weak CA_i*$Influence_i$					−1.56	2.55		
$Advise_i$							0.86	1.01
$Control_{ij}$*$Advise_i$							1.21	2.09
$Control_{ij}$*Strong CA_i*$Advise_i$							−1.48	2.58
$Control_{ij}$*Weak CA_i*$Advise_i$							−3.26	2.90

(Intercept)	7.50**	0.38	7.53**	0.38	7.53**	0.38	7.52**	0.38
Random-effects intercept for subjects (82 levels)								
Estimated *SD*	1.79		1.80		1.80		1.80	
Random-effects slope for $Control_{ij}$ within subjects (82 levels)								
Estimated *SD*	1.82		1.82		1.85		1.84	
R^2	0.79		0.79		0.79		0.79	
BIC	9525.10		9525.40		9528.10		9527.30	

* $p < 0.05$, ** $p < 0.005$ (uncorrected *p* values).

Note. The dependent variable is the chosen level by subject *i* in trial *j*. The HPRS subscale scores were normalized and mean-centered across subjects. The remaining predictors are as described in S 2. The models include a random-effects intercept for each subject and a random-effects slope for $Control_{ij}$ within subjects. The subject group without any peer information served as reference group. Sample size N = 82 subjects. Strong CA, subject group who observed a strongly control-averse peer; Weak CA, subject group who observed a weakly control-averse peer. HPRS subscales: Emotion, Emotional response toward restricted choices; Compliance, Reactance to compliance; Influence, Resisting influence from others; Advice, Reactance toward advice and recommendations.
